# Role of the Deubiquitylating Enzyme DmUsp5 in Coupling Ubiquitin Equilibrium to Development and Apoptosis in *Drosophila melanogaster*

**DOI:** 10.1371/journal.pone.0120875

**Published:** 2015-03-25

**Authors:** Levente Kovács, Olga Nagy, Margit Pál, Andor Udvardy, Octavian Popescu, Péter Deák

**Affiliations:** 1 Department of Genetics, University of Szeged, Szeged, Hungary; 2 Institute of Biochemistry, Biological Research Centre, Hungarian Academy of Sciences, Szeged, Hungary; 3 Molecular Biology Center, Interdisciplinary Research Institute on Bio-Nano-Sciences, Babes-Bolyai University, Cluj-Napoca, Romania; St. Georges University of London, UNITED KINGDOM

## Abstract

Protein ubiquitylation is a dynamic process that affects the function and stability of proteins and controls essential cellular processes ranging from cell proliferation to cell death. This process is regulated through the balanced action of E3 ubiquitin ligases and deubiquitylating enzymes (DUB) which conjugate ubiquitins to, and remove them from target proteins, respectively. Our genetic analysis has revealed that the deubiquitylating enzyme DmUsp5 is required for maintenance of the ubiquitin equilibrium, cell survival and normal development in Drosophila. Loss of the *DmUsp5* function leads to late larval lethality accompanied by the induction of apoptosis. Detailed analyses at a cellular level demonstrated that *DmUsp5* mutants carry multiple abnormalities, including a drop in the free monoubiquitin level, the excessive accumulation of free polyubiquitins, polyubiquitylated proteins and subunits of the 26S proteasome. A shortage in free ubiquitins results in the induction of a ubiquitin stress response previously described only in the unicellular budding yeast. It is characterized by the induction of the proteasome-associated deubiquitylase DmUsp14 and sensitivity to cycloheximide. Removal of *DmUsp5* also activates the pro-apoptotic machinery thereby resulting in widespread apoptosis, indicative of an anti-apoptotic role of DmUsp5. Collectively, the pleiotropic effects of a loss of DmUsp5 function can be explained in terms of the existence of a limited pool of free monoubiquitins which makes the ubiquitin-dependent processes mutually interdependent.

## Introduction

Ubiquitylation is the posttranslational modification of target proteins with an ubiquitin monomer or a polyubiquitin chain [1]. It directly affects protein turnover and basic intracellular processes, including cell cycle regulation and programmed cell death [2, 3]. Ubiquitin can be linked to proteins via an isopeptide bond between an internal lysine of the target protein and the terminal glycine residue of ubiquitin. Polyubiquitin chains are generated by ubiquitin polymerization through isopeptide bonds, most frequently between an internal lysine residue of the target protein and the terminal glycine of an incoming ubiquitin monomer [4]. Ubiquitylation is catalyzed by an enzyme cascade involving at least three different enzymes, with ubiquitin-activating (E1), ubiquitin-conjugating (E2), and ubiquitin ligase (E3) activities [5, 6]. Polyubiquitylation most frequently targets proteins for proteasomal degradation, but other, nondegradative roles are also well known [7, 8].

Apoptosis or programmed cell death is a key physiological process that is involved in shaping the development of multicellular organisms [9]. It is initiated by different death stimuli and culminates in the activation of a cascade of cysteine proteases, called caspases, which execute destruction. Since nearly all eukaryotic cells constitutively express all components of the apoptotic machinery, caspase activation must be regulated by a very precise and sensitive mechanism, if untimely cell death is to be prevented. In the course of the past decade, it has become firmly established that ubiquitylation has a major role in regulating apoptosis. Key apoptotic regulators, the inhibitor of apoptosis proteins (IAPs), are themselves E3 ubiquitin ligases, and the availability and abundance of the main pro- and anti-apoptotic proteins, including caspases, IAPs and IAP antagonists, are regulated by ubiquitin-mediated protein degradation [10]. More recently, nondegradative ubiquitylation, other E3 ligases and certain deubiquitylating enzymes have been reported to have roles in apoptosis [11, 12, 13], and it is conceivable that more ubiquitin pathway components will be linked to apoptosis.

Like other posttranslational modifications, ubiquitylation is a dynamic and reversible process in which deubiquitylating enzymes or DUBs counteract ubiquitin ligases by removing covalently linked ubiquitins from substrate proteins [14]. The DUBs are structurally diverse isopeptidases that specifically cleave ubiquitin conjugates at the ubiquitin carboxyl end. The activities of DUBs include the removal of intact ubiquitin monomers and polyubiquitin chains from conjugates, the disassembly of unanchored polyubiquitin chains to give intact monomers, and the processing of inactive ubiquitin precursor fusion proteins [15]. It has been well established that protein ubiquitylation is regulated by the coordinated action of ubiquitin ligases and deubiquitylating enzymes. This and the quite large number of known DUBs suggest the importance of deubiquitylation, perhaps because it permits a further layer of regulation in the ubiquitin-mediated biological processes, such as apoptosis.

In general, more is known about the biochemical and structural properties of DUBs than about their biological function. Reports relating to their physiological significance have mostly involved unicellular organisms and cell lines, although systematic functional analyses of DUBs in genetically tractable, intact multicellular organisms have also started to emerge [16, 17]. The biochemically best known DUB is Ubp14 from budding yeast and its USP5 or isopeptidase T ortholog from human cell lines [18, 19]. *In vitro* studies have revealed that this enzyme disassembles free polyubiquitin chains, most frequently liberated from ubiquitylated proteins just before proteasomal degradation [5, 20]. Deletion of the *UBP14* gene has been found to result in an accumulation of free polyubiquitin chains and the inhibition of proteasomal degradation [19]. Suppression of the human *USP5* gene also causes the accumulation of unanchored polyubiquitins and enhances the intracellular level of p53 [21]. Another study has demonstrated that USP5 is required for the efficient repair of DNA double-strand breaks in HeLa cells [22].

Annotation of the Drosophila genome sequence revealed that the *CG12082* gene encodes a protein with a similar aminoacid sequence to Ubp14/USP5 and it was later confirmed that it is an essential gene [23]. To date, only roles in activating apoptosis and the JNK pathway in the developing eye have been described for the Drosophila Ubp14/USP5 protein [24]. The heterologous complementation experiments reported here demonstrated that the protein encoded by the *CG12082* gene is a functional ortholog of the yeast/human Ubp14/USP5 protein, and a detailed phenotypic analysis of the partial and complete loss of function mutants is presented. Multiple abnormalities are revealed that lead to the induction of a phenomenon previously described only in the unicellular budding yeast: the ubiquitin stress response. The removal of *DmUsp5* additionally activates the pro-apoptotic machinery that results in widespread apoptosis in imaginal discs and the larval brain, pointing to a general anti-apoptotic role of DmUsp5. Apoptosis probably occurs through the downregulation of DIAP1, overexpression of which partially rescues both the lethal and apoptotic phenotypes of *DmUsp5* mutants. In summary, the phenotypic analysis presented here clearly indicates the importance of DmUsp5 in the normal progression of the ubiquitin cycle, and how this links ubiquitin-dependent processes such as protein degradation, apoptosis and development.

## Results

### Disruption of *CG12082* results in late larval and pupal lethality and widespread apoptosis

**Fig 1:**
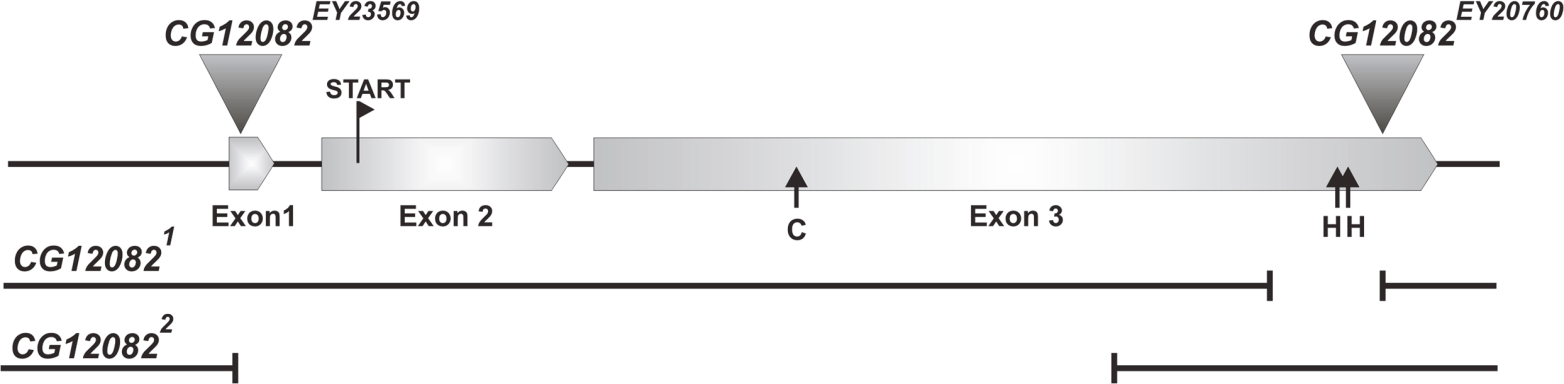
Schematic representation of *CG12082* P element insertion and deletion alleles. Triangles indicate the sites of P element insertions, while broken lines mark deleted segments in newly isolated mutants. Black arrows indicate positions of catalytic cysteine (**C**) and histidine (**H**) codons.

In a screen of transgenic RNA interference (RNAi) lines specific for genes coding for putative deubiquitylating enzymes, we used the Act5C-Gal4 and da-Gal4 general drivers for gene silencing and determined the resulting phenotypes. We identified several lines with a late lethal phenotype accompanied by widespread apoptosis in imaginal discs. Since silencing of the *CG12082* gene resulted in one of the most outstanding apoptotic phenotypes, we decided to further characterize this. For this purpose, we obtained independent transgenic RNAi lines (*CG12082* ^*v17567*^, *CG12082* ^*JF02163*^ and *CG12082* ^*NIG*.*12082R-2*^) and P element insertion alleles (*CG12082* ^*EY20760*^ and *CG12082* ^*EY23569*^), and isolated new alleles of *CG12082* (*CG12082* ^*1*^ and *CG12082* ^*2*^) by P element remobilization (Fig. 1). These lines form an allelic series with expression phenotypes ranging from basically wild type (*CG12082* ^*EY23569*^) through different hypomorphs to functional (*CG12082* ^*1*^) and true null (*CG12082* ^*2*^) alleles (Table 1). They influence development to extents depending on their degree of expression/loss of function, leading to viable, pupal or third-stage larval (L3) lethality (Table 1 and S1 Fig.). The lethal phenotype of the null mutants could be rescued by moderate expression of the wild type *CG12082* sequence (Table 2).

**Table 1:** Lethality of *CG12082* and *DmUsp14* alleles.

Genotype	Gene dose	Lethal phase			
		L3 larva	Pupa	Pharate adult	Viable
Oregon-R	Wild type			5%	95%
*da>CG12082* ^*v17567*^	Weak hypomorph	20%	80%		
*da>CG12082* ^*NIG*.*12082R-2*^	Hypomorph	30%	70%		
*da>CG12082* ^*JF02163*^	Strong hypomorph	70%	30%		
*CG12082* ^*EY23569*^	Wild type			3%	97%
*CG12082* ^*EY20760*^	Functional null	91%			
*CG12082* ^*1*^	Functional null	93%			
*CG12082* ^*2*^	True null	96%			
*da>DmUsp14* ^*v110227*^	Hypomorph			6%	94%
*DmUsp14* ^*f00779*^	Strong hypomorph			7%	93%^a^

^a^ Sterile males

**Table 2:** Transgenic rescue of *DmUsp5* mutants.

Genotype	Lethal phase			
	L3 larva	Pupa	Pharate adult	Viable
Oregon-R			5%	95%
*DmUsp5* ^*1*^	93%			
*DmUsp5* ^*2*^	96%			
*da>GFP-DmUsp5; DmUsp5* ^*1*^			24%	76%
*da>FLAG-DmUsp5; DmUsp5* ^*2*^			14%	86%
*da>UAS-DmUsp5; DmUsp5* ^*2*^		51%	49%	

**Fig 2:**
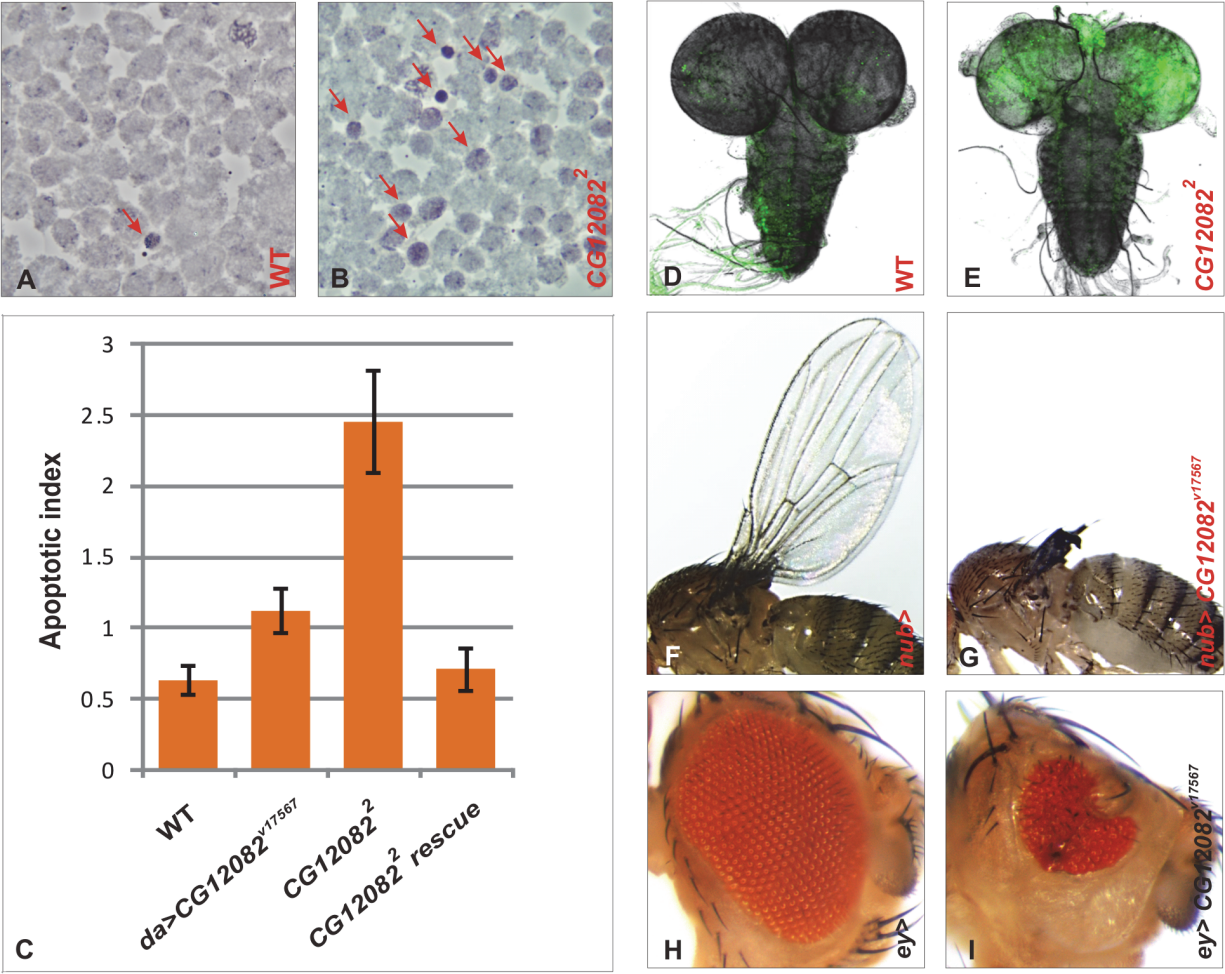
Loss of *CG12082 function* leads to elevated apoptosis in larval brain and imaginal discs. Wild type (A) and *CG12082* ^*2*^ (B) larval brain squashes were stained with orcein and investigated under a phase contrast microscope. Red arrows indicate small, rounded and pyknotic cells. Apoptotic index (C) was defined as the mean number of these cells in an optical field. Apoptotic indexes of wild type (WT), *CG12082* knock down (*da>CG12082* ^*v17567*^), *CG12082* null mutant (*CG12082* ^*2*^) and *CG12082* ^*2*^ rescue (*da>FLAG-CG12082*, *CG12082* ^*2*^) were defined. Each column represents the mean of 7–9 preparations. On average, 15–25 optical fields per preparations were scored. Acridine orange staining also marked dying cells in wild type (D) and *CG12082* null mutant (E) larval brains. Wing specific knock down of *CG12082* (G) resulted in reduced wings, while eye specific *CG12082* silencing (I) led to rough eye phenotype. Animals expressing the wing specific *nub-GAL4* (F) or *ey-GAL4* (H) driver alone, served as controls.

To elucidate the cause of the lethality observed, these mutants were examined for developmental and tissue defects. Orcein, acridine orange, and anti-activated caspase-3 immunostaining of larval brains and imaginal discs from strong hypomorph and null alleles revealed widespread apoptosis. Squash preparations of *CG12082* mutants (Fig. 2B) proved to contain many more small rounded and pyknotic cells as compared with the wild types (Fig. 2A). Similarly, the *CG12082* mutants exhibited a much higher level of apoptosis, as indicated by larger numbers of acridine orange- and activated caspase-3 positive cells (Fig. 2E and S2 Fig.) than in the wild type controls (Fig. 2D and S2 Fig.). All of the small pyknotic cells showed high expression of activated caspase-3 (S2 Fig. inset). Similarly to the lethal phenotype, the apoptotic phenotype could also be rescued by moderate expression of the wild type *CG12082* sequence (Fig. 2C).

Loss of *CG12082* function was further examined at a tissue-specific level in the developing eye and wing by using ey-GAL4 and nub-GAL4 drivers, respectively. The eye and wing-specific silencing of *CG12082* RNAi lines led to severely disrupted eyes and wings. The ey-GAL4-driven silencing resulted in rough and small (occasionally missing) eyes (Fig. 2I), while the nub-GAL4-specific silencing produced uninflated vestigial or missing wings (Fig. 2G). Since these phenotypes are very similar to those induced by apoptosis [25, 26, 27], we conclude that they are likely to result from cell death.

### The *CG12082* gene codes for a functional ortholog of the deubiquitylating enzyme Ubp14/USP5 in Drosophila

**Fig 3:**
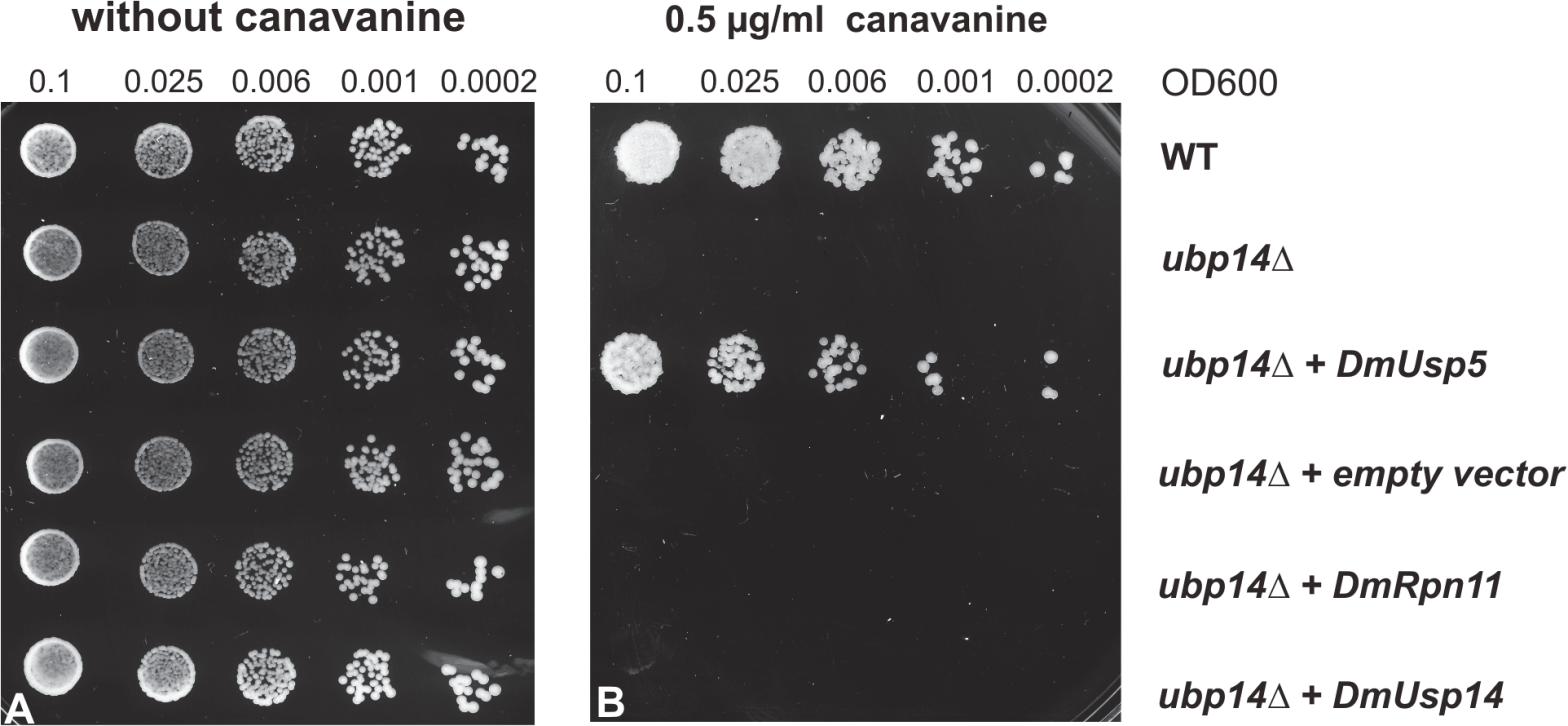
Expression of *CG12082* rescues the canavanine-sensitive phenotype of budding yeast *ubp14Δ* mutant cells. Wild type, *ubp14Δ* mutant and transformed *ubp14Δ* mutant cells carrying the indicated constructs were plated and grown on SD-dropout plates (A) or on SD-dropout plates supplemented with 0.5 μg/ml canavanine (B). Cells were plated in linear dilution series, each drop containing a quarter of the number of cells relative to the previous one. The plates were incubated at 30°C for 5 days.

On the use of FlyBase prediction data and BLAST analysis, the predicted protein encoded by the *CG12082* gene exhibited 43.8% and 65.1% overall similarity to yeast deubiquitylase Ubp14 and human deubiquitylase USP5, respectively. Further, the topology of its conserved domains, including UBP-type ZnF, USP, UBA1 and UBA2, corresponded closely to that found in the yeast Ubp14 and human USP5 proteins [24]. For experimental confirmation of their functional relatedness, heterologous complementation experiments were performed in which the protein encoded by *CG12082* could function as an Ubp14 substitute in budding yeast cells lacking their endogenous Ubp14 function. In these experiments, we introduced a CG12082 expression construct (*pVT102U-DmUsp5*) under the control of a constitutively active alcohol dehydrogenase promoter into ubp14Δ cells and tested for complementation of their canavanine-sensitive proliferation defect [19]. As illustrated in Fig. 3A, ubp14Δ cells grow as well as wild type cells on normal medium, but are unable to do so on a medium supplemented with 0.5 μg/ml canavanine (Fig. 3B). The ability of ubp14Δ cells to grow in the presence of canavanine was induced by the expression of the *CG12082* gene product (Fig. 3B). In similar experiments, the canavanine sensitivity of ubp14Δ cells could not be suppressed by the introduction of either the empty vector (*pVT102U*) or other DUBs (*pVT102U-DmRpn11* and *pVT102U-DmUsp14*). Since the human USP5/isoT also functioned as a Ubp14 substitute in yeast cells [19], these results suggest that the gene product of *CG12082* is a functional ortholog of the deubiquitylating enzyme Ubp14/USP5/isoT in Drosophila; we therefore renamed it DmUsp5.

### Loss of *DmUsp5* leads to the accumulation of free and conjugated polyubiquitin chains and a reduced level of free monoubiquitins

**Fig 4:**
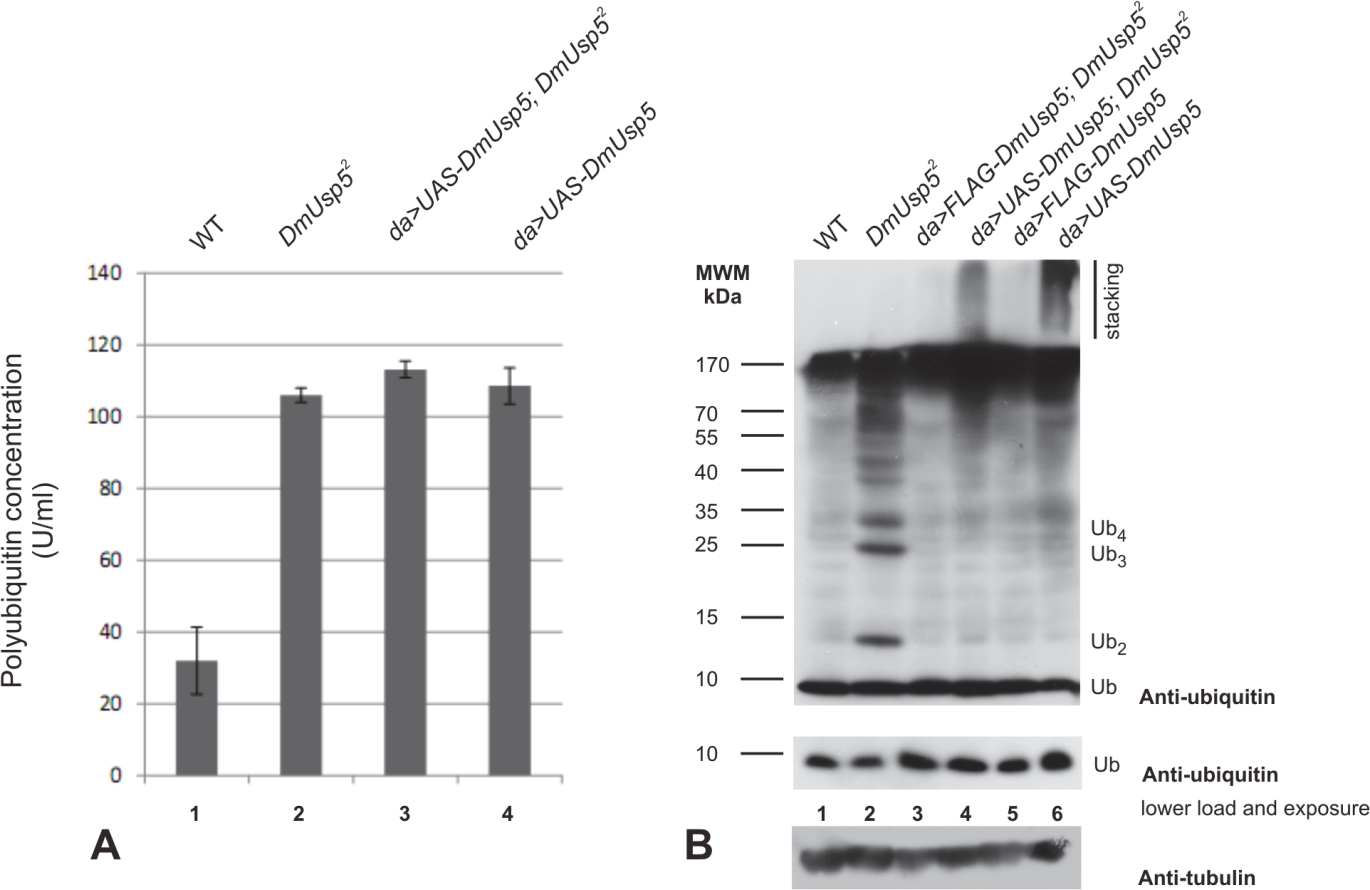
Accumulation of polyubiquitin species and reduction of monoubiquitin level in *DmUsp5* mutant. (A) Cyclex polyubiquitin-specific ELISA kit was used to measure the polyubiquitin concentration in third-stage larval protein extracts. Polyubiquitin levels were determined in wild type (column 1), *DmUsp5* null mutant (column 2) and animals strongly overexpressing *DmUsp5* either in mutant (column 3) or in wild type (column 4) background. (B) Third-instar larval protein extracts were separated on a 12% SDS-PAGE gel, blotted onto a PVDF membrane and immunostained with a polyclonal anti-ubiquitin primary antibody. Samples were prepared from wild type (lane 1), *DmUsp5* mutant (lane 2), animals moderately overexpressing *DmUsp5* either in mutant (lane 3) or in wild type (lane 5) background and animals strongly overexpressing *DmUsp5* either in mutant (lane 4) or in wild type (lane 6) background. Immunoblotting with anti-tubulin monoclonal antibody served as a loading control.

Several *in vitro* studies have shown that the main substrate of human USP5 is free polyubiquitin chains liberated from proteasome-bound polyubiquitylated proteins by proteasome-associated DUB, Rpn11[18, 28, 29]. In line with this, the loss of USP5 in yeast and in a human melanoma cell line led to the accumulation of free polyubiquitin chains [19, 21]. To investigate the molecular changes behind the observed phenotypes, a polyubiquitin-specific ELISA kit was used to determine possible changes in polyubiquitin level. Null alleles of *DmUsp5* caused an increase of ∼ 6-fold in the concentration of polyubiquitins as compared with the wild type control (Fig. 4A). Further Western blot analysis revealed that the polyubiquitin level elevation was due mainly to the accumulation of low and high molecular weight ubiquitin derivatives (Fig. 4B). The first group appeared as a ladder of 4–5 bands with molecular weights corresponding to those of free ubiquitin di-, tri-, tetra-, penta- and hexamers. The second group formed a dense smear of high molecular weight bands, most probably reflecting polyubiquitylated proteins.

Normally only a small proportion of the intracellular ubiquitin is in monomeric form [30, 31], and this monoubiquitin pool therefore has to be refilled constantly by DUBs [32, 33]. As shown above, in the *DmUsp5* mutants a significant proportion of the ubiquitin is trapped in conjugated form in free polyubiquitins and polyubiquitylated proteins, and it was therefore conceivable that the monoubiquitin pool becomes depleted. Repetition of the Western blot analysis under nonsaturating conditions revealed a lower free monoubiquitin concentration in the *DmUsp5* mutants relative to the wild type control (Fig. 4B, lanes 1 and 2). The abnormal accumulation of free polyubiquitins and polyubiquitylated proteins and the drop in free monoubiquitins were not seen when the wild type *DmUsp5* sequence was moderately expressed in the same mutant background (Fig. 4B, lane 3). These results demonstrate that, similarly as for yeast and human cell lines, DmUsp5 is required for the disassembly of free polyubiquitins and for the uninterrupted degradation of polyubiquitylated proteins in Drosophila. Moreover, the ubiquitin-recycling activity of DmUsp5 must reflect a major monoubiquitin supply route.

Interestingly, the UAS/GAL4-driven strong overexpression of the wild type DmUsp5 appeared to be detrimental to the animals and caused temperature-sensitive lethality in late development (Table 3). A polyubiquitin-specific ELISA experiment (Fig. 4A) and Western blot analysis of protein extracts revealed a significant accumulation of polyubiquitylated proteins in the cells of DmUsp5-overexpressing flies (Fig. 4B). Even more unusually, a well-detectable fraction of this ubiquitylated species could not enter the separating gel, but remained trapped and displayed smeary staining in the stacking gel (Fig. 4B). This species behaves similarly to protein aggregates with low solubility [34]. These observations indicate that the overexpression of DmUsp5 also interferes with proteasomal degradation. The possible mechanism will be discussed below.

**Table 3:** Temperature-dependent lethality of DmUsp5 overexpression.

Genotype	Lethal phase			
	L3 larva	Pupa	Pharate adult	Viable
Oregon-R at 25°C			5%	95%
*da>UAS-DmUsp5* at 18°C		34%	62%	4%
*da>UAS-DmUsp5* at 25°C		89%	11%	
*da>UAS-DmUsp5* at 29°C	10%	90%		

### Loss of *DmUsp5* leads to the induction of the proteasome-associated DUB DmUsp14 and the upregulation of proteasome subunits

Physiological consequences of a low ubiquitin level have been studied only in the budding yeast *Saccharomyces cerevisiae*. A decline in free monoubiquitin levels was demonstrated to lead to induction of the ubiquitin stress response [35, 36]. A hallmark of this ubiquitin stress is the induction of the proteasome-associated DUB Ubp6 [36]. It is thought that Ubp6 associates with the proteasome, inhibits its proteolytic activity and catalyzes the gradual removal of monoubiquitins from the distal end of protein-conjugated polyubiquitin chains [35, 37], thereby contributing to replenishment of the free monoubiquitin pool. Since the loss of the DmUsp5 function generates a low ubiquitin state, it provides an opportunity to demonstrate the existence of a similar phenomenon in Drosophila. We therefore looked at the expression level of the Drosophila ortholog of *Ubp6* in our mutants.

In FlyBase, the *CG5384* gene codes for a putative DUB. Comparison of its aminoacid sequence with those of known yeast and human DUBs revealed a high sequence similarity and an identical domain topology to those of yeast UBP6 and the human USP14 proteins (S3 Fig.). The protein coded by the *CG5384* gene is therefore regarded as the Drosophila ortholog of these proteins and we renamed it DmUsp14. Silencing the *DmUsp14* gene by RNA interference did not produce any visible phenotype, but a PBac insertion allele showed male sterility (Table 1).

**Fig 5:**
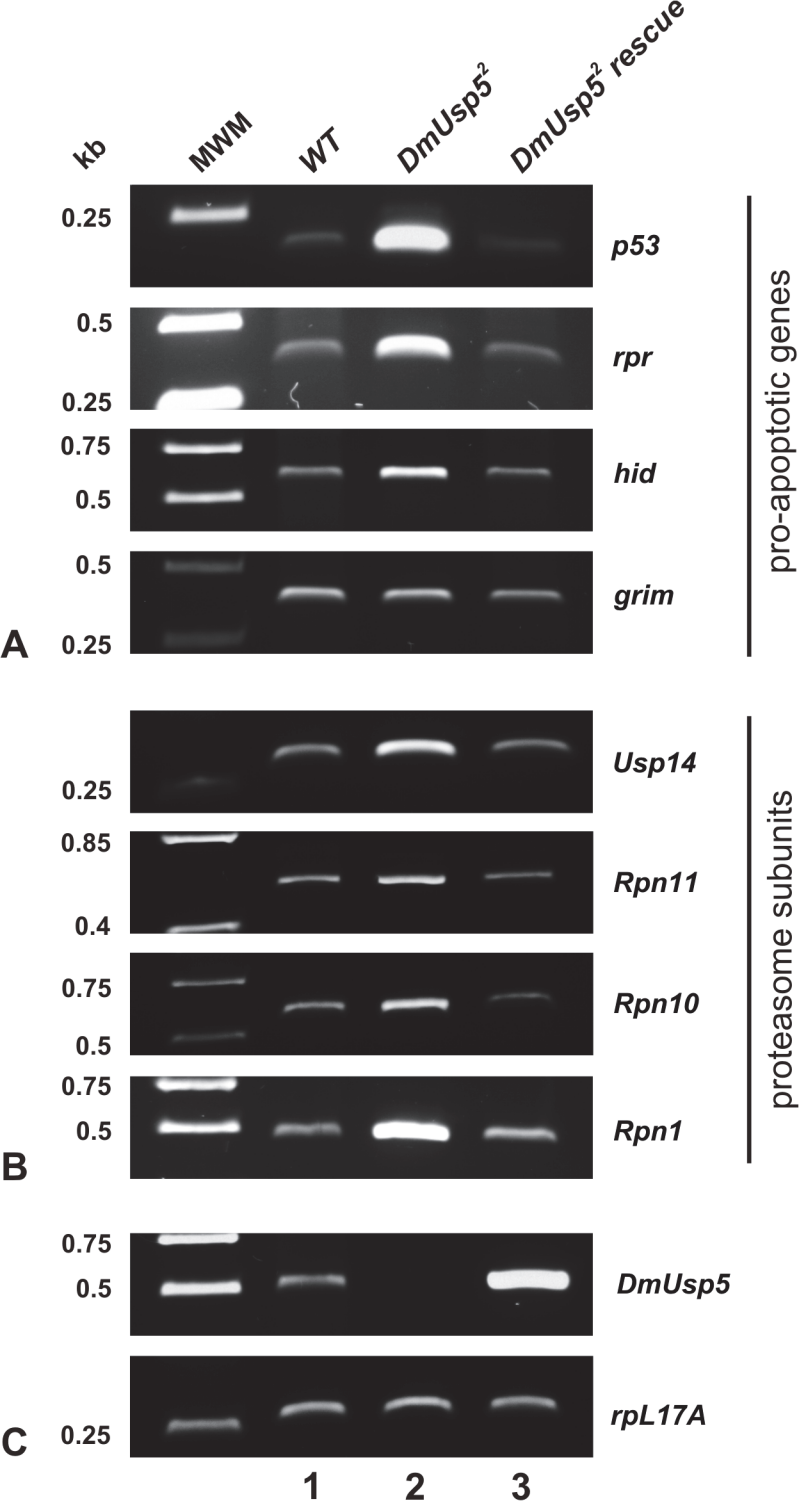
Overexpression of pro-apoptotic genes and proteasome subunits in the *DmUsp5* mutant. Total cDNA samples were prepared from wild type (lane1), *DmUsp5* null mutant (lane 2) and *DmUsp5* ^*2*^ rescue (lane 3) third-stage larvae. Semiquantitative PCRs were performed using primers specific to mRNAs of pro-apoptotic (panel A) and proteasome subunit (panel B) genes. *DmUsp5* transcript specific primers were used to determine *DmUsp5* expression, while *rpL17* served as a loading control (panel C).

Semiquantitative RT-PCR was used to determine the *DmUsp14* expression levels in loss of function *DmUsp5* mutants and in wild type flies. As the expression of *DmUsp14* was upregulated in the absence of DmUsp5 (Fig. 5B), we propose that the accumulation of free polyubiquitin chains eventually leads to a free monoubiquitin deficiency and to the induction of ubiquitin stress in Drosophila.

The accumulation of polyubiquitylated proteins in *DmUsp5* mutants is reminiscent of the phenotype of essential proteasome subunit mutants. It was earlier reported that deletion of the essential Rpn10 proteasomal subunit resulted in a substantial accumulation of all the proteasomal subunits [38, 39], a phenomenon similar to that described in yeast as a proteasome stress response [36]. To detect the expression of proteasome subunits, total RNA was isolated from *DmUsp5* mutant and wild type animals and the expressions of *Rpn1-*, *Rpn10-* and *Rpn11*-specific transcripts were monitored by semiquantitative RT-PCR. Each of these genes revealed an elevated expression in the null mutant as compared with the wild type (Fig. 5B). The detected overexpression of these genes was cured by moderate expression of the wild type *DmUsp5* sequence in the mutant background. These results demonstrate a coordinated upregulation of proteasome subunits in the absence of DmUsp5, and hence the induction of proteasome stress.

### *DmUsp5* mutants show cycloheximide sensitivity

**Fig 6:**
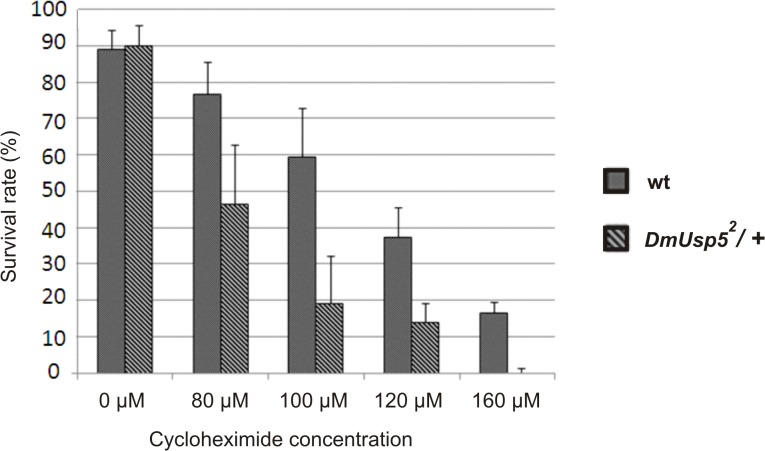
*DmUsp5* mutant heterozygotes are sensitive to cycloheximide. Synchronized first-instar larvae were collected from wild type (wt) and *DmUsp5* mutant heterozygotes (*DmUsp5* ^*2*^/+). Larvae were treated with the indicated concentrations of cycloheximide solution. The number of survival adults were counted. Each column represents the mean of four independent treatments.

Ubiquitin depletion in yeast sensitizes cells to different chemicals, including translational inhibitors such as cycloheximide [40, 41], and is regarded as a distinctive feature of ubiquitin stress. To support our assumption that loss of the Usp5 function gives rise to ubiquitin stress, the sensitivity of *DmUsp5* mutants to cycloheximide was analyzed. Since the loss of *DmUsp5* leads to lethality, we used *DmUsp5* ^*2*^/+ heterozygotes for this test. The data presented in Fig. 6 demonstrate that even the partial reduction of DmUsp5 in the heterozygotes creates a marked dose-dependent sensitivity to cycloheximide relative to the wild type (Fig. 6).

This is therefore a dominant phenotype, which further underlines the ubiquitin deficiency and the ubiquitin stress in the cells of *DmUsp5* mutants.

### Pro-apoptotic genes are activated in *DmUsp5* mutants

The core of the apoptotic machinery in Drosophila contains initiator and effector caspases, inhibitor of apoptosis proteins (IAPs) and pro-apoptotic proteins such as p53, Reaper, Hid and Grim (the last three frequently mentioned as the RHG proteins) [42]. Following a cytotoxic stimulus, apoptosis is induced by the transcriptional activation of P53 and the RHG coding genes. The RHG proteins competitively bind to Drosophila IAP1 (DIAP1) and, by antagonizing its caspase inhibitory function, promote caspase-dependent cell killing [10]. To determine whether DmUsp5 loss leads to apoptosis through this pathway, we followed the expression of the RHG genes by semiquantitative RT-PCR and quantitative real time PCR. Fig. 5A demonstrates that the expression of p53, Reaper and Hid is elevated in the *DmUsp5* mutant as compared with the wild type control. The highest induction in expression level was observed with p53 and Reaper. This higher expression returned close to the wild type level when DmUsp5 was moderately expressed in the mutant background. We did not detect a significant change in the expression of Grim (Fig. 5A). The expression profiles of these genes were confirmed by quantitative real time PCR measurements (S4 Fig.). These results suggest that the loss of DmUsp5 activates at least three members of the pro-apoptotic machinery and it must therefore act upstream of them.

### Overexpression of DIAP1 partially rescues both the lethal and the apoptotic phenotypes of *DmUsp5* mutants

**Fig 7:**
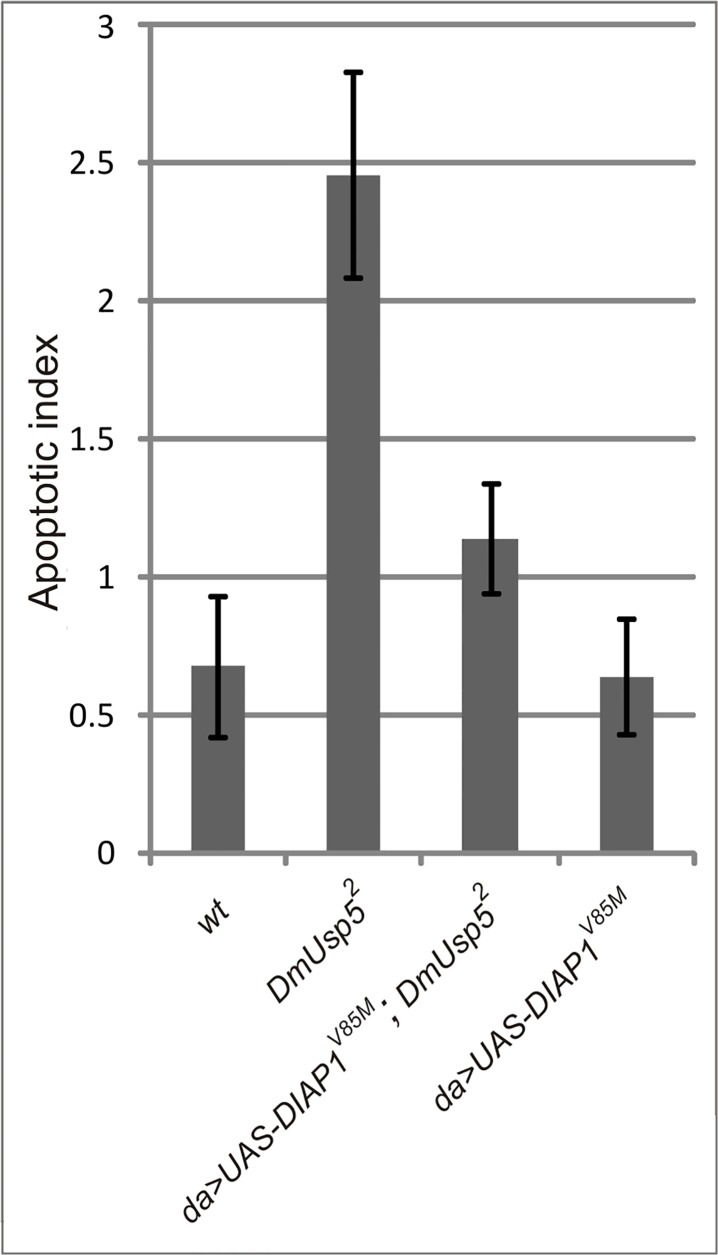
DIAP1 overexpression rescues the apoptotic phenotype of the *DmUsp5* mutant. Apoptotic indexes of wild type (wt), *DmUsp5* mutant (*DmUsp5* ^*2*^) and DIAP1 overexpressing animals either in mutant (*da>UAS-DIAP1* ^*V85M*^; *DmUsp5* ^*2*^) or in wilt type (*da>UAS-DIAP1* ^*V85M*^) background were determined as described in Fig. 2C. Each column represents the mean of 7–8 preparations.

Besides liberating caspases, Reaper can further augment apoptosis by downregulating DIAP1 through promoting its auto-ubiquitylation and through a general inhibitory effect on translation [43, 44]. We did not follow DIAP1 levels directly, but instead, overexpressed DIAP1 in the *DmUsp5* ^*2*^ null mutant and monitored its phenotypic effects. DIAP1 overexpression exhibited significant effects on both the lethal and the apoptotic phenotypes. DIAP1 overexpression shifted the late larval (L3) lethality of the *DmUsp5* ^*2*^ mutant to the pupal stage, while the overexpression itself had no effect on the survival and fertility of the control flies (Table 4). This experiment was followed up by determining the frequency of apoptotic cells on orcein-stained larval brain and imaginal disk preparations. DIAP1 overexpression proved to reduce the apoptotic index of *DmUsp5* ^*2*^ by about 50% (Fig. 7). These findings are consistent with the notion that DIAP1 is downregulated in *DmUsp5* mutants.

**Table 4:** Effects of DIAP1 overexpression on *DmUsp5* ^*2*^ lethal phase.

Genotype	Lethal Phase			
	L3 larva	Pupa	Pharate adult	Viable
Oregon-R			5%	95%
*da>UAS-DIAP1* ^*V85M*^			6%	94%
*DmUsp5* ^*2*^	96%			
*da>UAS-DIAP1* ^*V85M*^; *DmUsp5* ^*2*^	22%	78%		

## Discussion

It has become firmly established that a large variety of essential intracellular processes are regulated by ubiquitylation. Ubiquitin is a rather abundant polypeptide that exists in eukaryotic cells as free monomer, free polymer and protein-conjugated mono- or polyubiquitin. For the normal cell physiology, these forms are maintained in dynamic equilibrium, in which ubiquitin cycles between the free and conjugated states [45, 46], mediated by the opposing processes of ubiquitylation and deubiquitylation. It has been demonstrated that, at any given time, in most tissues only a small proportion of the total cellular ubiquitin content is available as free monomer, while the majority is anchored to proteins [30, 31, 45]. Because of the high demand for a limited pool of free ubiquitins, normal cell functioning requires a continuous production of an adequate amount of free monoubiquitins. Active free monoubiquitins are generated by the deubiquitylating enzymes from inactive precursor fusion products and by the recycling of previously used ubiquitins [32, 33].

In this study, we have demonstrated in a heterologous complementation experiment that the Drosophila *CG12082* gene encodes the functional ortholog of the yeast Ubp14 and the human USP5/isopeptidase T deubiquitylases. Moreover, *in vivo* experimental data are presented that reveal an important role of this DUB in the regulation of intracellular ubiquitin levels in *Drosophila melanogaster*. Loss of function *DmUsp5* mutants display huge increases in the unconjugated and conjugated polyubiquitin levels and, simultaneously, a significant reduction in the free monoubiquitin concentration. The accumulation of free polyubiquitins in the mutant is consistent with *in vitro* and *in vivo* data pointing to the main function of this enzyme as being the disassembly of free polyubiquitin chains [5, 19, 20, 21]. The accumulation of polyubiquitylated proteins can be explained by the fact that free polyubiquitins bind to proteasome-associated polyubiquitin receptors with high affinity [47, 48]. In excess, they competitively inhibit the targeting of polyubiquitylated substrates to the proteasome, resulting in the observed accumulation of polyubiquitylated proteins. The coordinated upregulation of proteasome subunits in *DmUsp5* mutants, the hallmark of the proteasome stress response [36], supports the inhibition of proteasome activity.

Although we detected a reduction of the level of free monoubiquitin in *DmUsp5* mutants, the question arises as to whether this drop is significant enough to be below the critical physiological threshold. The induction of the ubiquitin stress response in *DmUsp5* mutants is a direct indication that even this moderate drop in monoubiquitin concentration has serious physiological consequences. All the critical features of ubiquitin stress described in the yeast are characteristic properties of the *DmUsp5* mutants: loss of the DmUsp5 function leads to a dominant cycloheximide sensitivity in Drosophila (Fig. 6), with a marked increases in the expression levels of both the *DmUsp14* gene and the genes of several proteasomal subunits (Fig. 5B). Taken together, these observations suggest that DmUsp5 has a major role in maintaining the free monoubiquitin pool in Drosophila. When its function is impaired, the free monoubiquitin shortage may compromise ubiquitin homeostasis.

Another characteristic feature of *DmUsp5* mutants is the abnormally high incidence of apoptosis in the imaginal tissues which is the most probable cause of the late larval lethality and could be directly linked to a free monoubiquitin shortage. Apoptosis is one of the ubiquitin-mediated pathways and is negatively regulated in a dynamic process by DIAP1 and protein degradation in Drosophila. DIAP1 ensures cell survival by regulating the abundance and stability of caspases and IAP antagonists through its E3 ubiquitin ligase activity. Ubiquitylation is therefore intrinsic to the anti-apoptotic activity of DIAP1 and it is quite conceivable that an ubiquitin shortage alone or together with an abnormally high level of polyubiquitins (free and conjugated) and crippled protein degradation may generate a death signal that activates the pro-apoptotic machinery, resulting in the abnormally high level of apoptosis observed in *DmUsp5* mutants. The elevated expression of the pro-apoptotic *p53* and some of the RHG genes in the absence of the DmUsp5 function support this idea. Although the molecular mechanism of apoptotic activation is not known, in the *DmUsp5* mutants it must take place upstream of the transcriptional activation of the RHG genes. Proteasome inhibition, which also leads to a free monoubiquitin shortage and eventually to apoptosis, was previously reported, to induce a shift in the distribution of conjugated ubiquitin from mainly nuclear to mainly cytoplasmic locations, and this coincided with a significant depletion of ubiquitylated histone H2A in the chromatin, together with chromatin remodeling [45]. It was also shown that this ubiquitin relocation was a direct effect of a competition for the limited pool of free monoubiquitins. Since changes in histone ubiquitylation are known to lead to major changes in gene expression [49], and histone H2A ubiquitylation is required for gene silencing [50], it is reasonable to conceive that a prolonged ubiquitin shortage could trigger expression of the RHG genes and thereby induce apoptosis during proteasome inhibition and in loss of function *DmUsp5* mutants too. This limited pool of free monoubiquitins links protein degradation to apoptosis and to development by making these ubiquitin-dependent processes mutually interdependent. The different pleiotropic effects indicate that DmUsp5 has a pivotal role in maintaining the free monoubiquitin pool.

During the course of the present manuscript preparation, an *in press* study appeared exploring the role of DmUsp5 in Drosophila development [51]. This reveals that DmUsp5 is essential for viability, and demonstrates that free and conjugated polyubiquitin chains accumulate in *DmUsp5* mutants, and that the levels of proteasome subunits are also elevated, findings with which our study fully agrees. It suggests an abnormal ubiquitin homeostasis behind these aberrations, though the ubiquitin stress and the apoptotic phenotype of the mutants were not analyzed or interpreted.

The fact that strong ectopic overexpression of the wild type DmUsp5 interfered with proteasomal degradation and development was somewhat surprising. The degree of overexpression was driver-, genomic location- and temperature-dependent (Fig. 4B, lanes 4–6). When the expression was driven by the da-GAL4 driver at different temperatures, the staining intensity of the high molecular weight smear of the ubiquitin species in both the stacking and separating gels was stronger at higher temperatures (S5 Fig.) and was directly proportional to the shift in the lethal phase of the animals: higher expression was associated with earlier lethality (Table 3). The electrophoretic behavior of the high molecular weight ubiquitin species was reminiscent of that of protein aggregates with low solubility [34]. A plausible explanation of this phenomenon could be that the huge amount of ectopically expressed DmUsp5 proteins overwhelms the protein-folding machinery of the cells, with the result that misfolded proteins appear which are prone to form different associations and aggregates [52]. Misfolded proteins become polyubiquitylated for proteasomal degradation, but it has emerged that aggregate-prone proteins are dangerous proteasome substrates. It has been demonstrated that protein aggregates inhibit the activity of proteasomes [53, 54]. The precise mechanism of the inhibition is not known, but it has been suggested that proteasomes could capture tangled proteins, but are then unable either to degrade or to release them, so that they become stalled [53]. Be that as it may, proteasome inhibition spells an abnormal ubiquitin cycle and a shortage in free ubiquitins, which explains the lethal effect of DmUsp5 overexpression.

## Materials and Methods

### Drosophila stocks and techniques

Fly stocks were raised on standard yeast/cornmeal/dextrose medium at 25°C. P element insertion and transgenic RNA interference lines were obtained from the Bloomington Drosophila Stock Center, the Vienna Drosophila Resource Center and NIG-Fly. All genetic markers used are described in FlyBase at http://flybase.org.

In order to determine the lethal phase, 200–400 first-instar homozygous and heterozygous larvae were collected and counted. Metamorphosis was staged according to Bainbridge and Bownes [55].

### P element remobilization

*DmUsp5* deletion alleles were generated by the P element remobilization technique [56]. Imprecise excision of *P{EPgy2}EY20760* generated the *usp5* ^*1*^ allele carrying a 275 bp deletion which eliminates the catalytic histidine box and UBA2 domain coding sequence. Imprecise excision of *P{EPgy2}EY23569* resulted in the *usp5* ^*2*^ null allele bearing a 2175 bp deletion eliminating the gene. Since both mutants exhibited the same lethal and apoptotic phenotype, we used the *usp5* ^*2*^ null allele for further biochemical characterization.

### Semiquantitative RT-PCR

Total RNA was isolated from 10 wandering third-instar larvae through use of a Tri Reagent extraction kit (Sigma-Aldrich, USA). RNA samples were treated with RQ1 RNase-Free DNase (Promega Corporation, USA). Reverse transcription was carried out with a Fermentas cDNA synthesis kit. Samples of cDNA were normalized to the *rpL17A* level, and the pro-apoptotic and proteasome subunit gene expressions were then determined by 20–25-cycle PCRs with the use of exon specific primers.

### Quantitative real time PCR

For quantitative real time PCR, 1 microgram of total RNA was reverse transcribed using RevertAid First Strand cDNA synthesis kit (Thermo Scientific) according to the manufacturer’s protocol. Real time PCR was performed using an ABI 7500 real time PCR system (Applied Biosystems) with SYBR Green chemistry, under the following conditions: 1 cycle of 95°C for 10 minutes, 40 cycles of 95°C for 15 second and 60°C for 1 minute. Primers were designed by the Clone Manager software. The C_t_ value for each mRNA was normalised to the *rpL17A* ribosomal RNA and *Actin 42A* RNA internal controls. The changes in expression levels of the examined genes were calculated by the ΔΔC_t_method. Primers for quantitative real time PCR:

*Actin42A* forward: GCGTCGGTCAATTCAATCTT

*Actin42A* reverse: AAGCTGCAACCTCTTCGTCA

*RpL17A* forward: GAGCCAAGAACCTGTACG

*RpL17A* reverse: CAGGCATGACCTTCTTCC

*p53* forward: CATCGAGGGCATGATTAAGG

*p53* reverse: CGTAGGCACGTTTCTTAAGG

*rpr* forward: AGGAGCAGCAGATCCTTC

*rpr* reverse: TCTTCCGGTCTTCGGATG

*hid* forward: ACCGACCAAGTGCTATACG

*hid* reverse: GCGGATACTGGAAGATTTGC

*grim* forward: GCGGTCAGAACAACGATG

*grim* reverse: GATTGAGCCTGCCTTTCC

### Cytological analysis

Brains from third-instar larvae were dissected for both orcein and acridine orange staining. Details of these staining protocols can be found in [57]. Preparations were analyzed under an Olympus BX51 upright microscope, or with an Olympus FV 1000 confocal microscope.

For immunohistochemistry, imaginal discs from late third instar larvae were dissected in PBS and fixed in 4% formaldehyde. Samples were washed in PBSTx and blocked in 1% BSA. Rabbit polyclonal Cleaved Caspase-3 Antibody (Cell Signaling Technology) was used in 1:200 dilution. The primary antibody was detected by Alexa Fluor 647 Goat Anti-Rabbit (Life Technologies) secondary antibody in 1:400 dilution. Pictures were taken by an Olympus FV10i confocal microscope.

### *DmUsp5* tagging and overexpression

The coding sequence of *DmUsp5* was cloned into the pUAST Drosophila expression vector between the XhoI/XbaI sites. The resulting *pUAST-DmUsp5* plasmid was verified by sequencing and injected into isogenized *w* ^*1118*^ embryos. Transformants were selected for the presence of the *w* ^*+mC*^ marker. Transgene localization was mapped to chromosomes by using a balancer line marked on the second and third chromosomes. Transgene expression was induced by the ubiquitous *da-Gal4* driver.

In order to generate tagged *DmUsp5*-expressing lines, the *DmUsp5* coding sequence was cloned into pENTR1A and recombined into pTFWattB (for N terminal FLAG tagging) or pTGWattB (for N terminal GFP tagging) expression vectors. The phiC31 integrase-mediated site-specific transformation resulted in transgene insertion into the *su(Hw)attP1* landing site on the third chromosome. The expression of the transgene was induced by the *da-Gal4* driver. The presence of *FLAG-DmUsp5* was verified by Western blotting, while the production and localization of *GFP-DmUsp5* were examined by confocal microscopy.

### Yeast heterologous complementation test

The yeast strains and vectors used in this work were kindly provided by Mark Hochstrasser. Standard rich and minimal yeast media and yeast techniques were used.

The *DmUsp5* encoding RE70722 and *DmUsp14* encoding LP08774 cDNA clones were ordered from DGRC. These coding sequences were cloned into the pVT102U yeast expression vector. The *pVT102U-DmUsp5*, *pVT102U-DmUbp6*, *pVT102U-DmRpn11* and the empty vector were transformed into the *UBP14*-deficient MHY840 (*MATα hisΔ200 leu2–3*, *112 ura3–52 lys2–801 trp1–1 ubp14Δ1*::*HIS3*) strain. Single Ura^+^ colonies were selected and then inoculated into 3 ml of rich YPAD media and incubated overnight at 30°C. The wild type MHY501 (*MATα hisΔ200 leu2–3*, *112 ura3–52 lys2–801 trp1–1*) strain served as control. Overnight cultures were diluted to OD_600_ = 0.1 and then four-fold serially diluted four times. 5 μl of each dilution was spotted onto nitrogen-rich, Arg-free SC media containing 0.5 μg/ml canavanine sulfate. Plates were incubated at 30°C for 5 days, after which photos were taken.

### SDS-PAGE and Western blots

Protein samples were prepared from third-instar larvae or pupae. Proteins were separated in a 12% resolving gel by SDS-PAGE. The protein load was optimized by Coomassie Brilliant Blue staining. Samples were blotted onto a PVDF membrane and immunostained with 1:5000 Polyclonal Rabbit Anti-Ubiquitin primary antibody (DAKO, Denmark) and 1:33000 anti-rabbit IgG-HRP secondary antibody (DAKO, Denmark). Samples were optimized using a mouse anti-beta-tubulin monoclonal antibody (clone ID: E7, DSHB, USA) in 1:1000 dilution. It was detected with goat anti-Mouse IgG-HRP secondary antibody (DAKO, Denmark) in 1:33000 dilution.

### Cycloheximide treatment

Cycloheximide powder (Sigma-Aldrich, USA) was dissolved in 34% EtOH to obtain a 17.6 mM stock solution. First-instar larvae were collected in vials containing 3.5 ml of standard Drosophila medium and fed with 60 μl of cycloheximide solution appropriately diluted in 34% EtOH. The feeding concentration was calculated to a final volume of 3.5 ml. Emerging adults were counted and statistically analyzed with the use of Microsoft Office Excel.

## Supporting Information

S1 Fig Expression of *CG12082* in transgenic RNA interference, P element insertion and deletion mutant alleles. Expression levels were determined by semiquantitative RT-PCR followed by agarose gel electrophoresis. Samples were normalized to *RpL17A* expression.
(TIF)

S2 Fig *CG12082* mutant imaginal discs show strong cleaved caspase-3 specific immunostaining. Leg discs of late L3 instar larvae from wild type (upper row) and *CG12082* ^*2*^ (lower row) were stained with DAPI (blue) and anti-cleaved caspase-3 antibody (red). Insets show individual cells at higher magnification.
(TIF)

S3 Fig DmUsp14 shares high sequence similarity and an identical domain topology with yeast Ubp6 and human USP14. Amino acid sequences were aligned using Align X tool of Vector NTI 10 software. Conserved and similar amino acids are highlighted by red and yellow backgrounds, respectively. Black underline indicates the ubiquitin-like (UBL) domain. Catalytic cysteine and histidine residues are marked by black arrows.
(PDF)

S4 Fig Pro-apoptotic genes are overexpressed in *DmUsp5* mutant. Total RNA was extracted from wild type and *DmUsp5* ^*2*^ larvae and reverse transcribed into cDNA. Quantitative real time PCR was performed on each pro-apoptotic gene normalized to *Actin42A* and *rpL17A* housekeeping genes. Columns represent the fold changes of gene expressions in *DmUsp5* ^*2*^ compared to wild type levels. Data represent mean and standard deviation of two independent experiments.
(TIF)

S5 Fig Overexpression of DmUsp5 leads to temperature-sensitive accumulation of high molecular weight ubiquitin species. Protein extracts were prepared from third-instar larvae overexpressing DmUsp5 at the indicated temperatures. Samples were separated in an 8% SDS-PAGE gel, blotted onto a PVDF membrane and immunostained with a polyclonal anti-ubiquitin primary antibody. The Western blot reveals a fraction of high molecular weight ubiquitin species trapped in the stacking gel.
(TIF)

## References

[1] Kerscher O, Felberbaum R, Hochstrasser M. Modification of proteins by ubiquitin and ubiquitin-like proteins. Annu Rev Cell Dev Biol. 2006; 22: 159–180. 16753028 10.1146/annurev.cellbio.22.010605.093503

[2] Bader M, Steller H. Regulation of cell death by the ubiquitin-proteasome system. Curr Opin Cell Biol. 2009; 21: 878–884. 10.1016/j.ceb.2009.09.005 19850458 PMC2818673

[3] Mocciaro A, Rape M. Emerging regulatory mechanisms in ubiquitin-dependent cell cycle control. J Cell Sci. 2012; 125: 255–263. 10.1242/jcs.091199 22357967 PMC3283867

[4] Hershko A, Ciechanover A. The ubiquitin system. Annu Rev Biochem. 1998; 67: 425–479. 9759494 10.1146/annurev.biochem.67.1.425

[5] Wilkinson KD, Tashayev VL, O'Connor LB, Larsen CN, Kasperek E, et al Metabolism of the polyubiquitin degradation signal: structure, mechanism, and role of isopeptidase T. Biochemistry. 1995; 34: 14535–14546. 7578059 10.1021/bi00044a032

[6] Pickart CM. Mechanisms underlying ubiquitination. Annu Rev Biochem. 2001; 70: 503–533. 11395416 10.1146/annurev.biochem.70.1.503

[7] Kaiser P, Flick K, Wittenberg C, Reed SI. Regulation of transcription by ubiquitination without proteolysis: Cdc34/SCF(Met30)-mediated inactivation of the transcription factor Met4. Cell. 2000; 102: 303–314. 10975521 10.1016/s0092-8674(00)00036-2

[8] Jentsch S, Rumpf S. Cdc48 (p97): a “molecular gearbox” in the ubiquitin pathway? Trends Biochem Sci. 2007; 32: 6–11. 17142044 10.1016/j.tibs.2006.11.005

[9] Steller H. Mechanisms and genes of cellular suicide. Science. 1995; 267: 1445–1449. 7878463 10.1126/science.7878463

[10] Broemer M, Meier P. Ubiquitin-mediated regulation of apoptosis. Trends Cell Biol. 2009; 19: 130–140. 10.1016/j.tcb.2009.01.004 19217783

[11] Ditzel M, Broemer M, Tenev T, Bolduc C, Lee TV, Rigbolt KT, et al Inactivation of effector caspases through nondegradative polyubiquitylation. 2008; Mol Cell 32:540–553. 10.1016/j.molcel.2008.09.025 19026784 PMC2713662

[12] Zaman MM, Nomura T, Takagi T, Okamura T, Jin W, Shinagawa T, et al Ubiquitination-deubiquitination by the TRIM27-USP7 complex regulates tumor necrosis factor alpha-induced apoptosis. 2013; Mol Cell Biol 33:4971–4984. 10.1128/MCB.00465-13 24144979 PMC3889550

[13] Yang CS, Sinenko SA, Thomenius MJ, Robeson AC, Freel CD, Horn SR, et al The deubiquitinating enzyme DUBAI stabilizes DIAP1 to suppress Drosophila apoptosis. Cell Death Differ. 2014; 21: 604–11. 10.1038/cdd.2013.184 24362437 PMC3950323

[14] Song L, Rape M. Reverse the curse—the role of deubiquitination in cell cycle control. Curr Opin Cell Biol. 2008; 20: 156–163. 10.1016/j.ceb.2008.01.012 18346885 PMC2387050

[15] Amerik AY, Hochstrasser M. Mechanism and function of deubiquitinating enzymes. Biochim Biophys Acta. 2004; 1695: 189–207. 15571815 10.1016/j.bbamcr.2004.10.003

[16] Chen X, Fischer JA. *In vivo* Structure/Function analysis of the Drosophila fat facets deubiquitinating enzyme gene. 2000; Genetics 156: 1829–1836. 11102377 10.1093/genetics/156.4.1829 PMC1461389

[17] Doelling JH, Yan N, Kurepa J, Walker J, Vierstra RD. The ubiquitin-specific protease UBP14 is essential for early embryo development in Arabidopsis thaliana. Plant J. 2001; 27: 393–405. 11576424 10.1046/j.1365-313x.2001.01106.x

[18] Hadari T, Warms JV, Rose IA, Hershko A. A ubiquitin C-terminal isopeptidase that acts on polyubiquitin chains. Role in protein degradation. Biol Chem. 1992; 267: 719–727. 1309773

[19] Amerik AY, Swaminathan S, Krantz BA, Wilkinson KD, Hochstrasser M. *In vivo* disassembly of free polyubiquitin chains by yeast Ubp14 modulates rates of protein degradation by the proteasome. EMBO J. 1997; 16: 4826–4838. 9305625 10.1093/emboj/16.16.4826 PMC1170118

[20] Falquet L, Paquet N, Frutiger S, Hughes GJ, Hoang-Van K, et al A human de-ubiquitinating enzyme with both isopeptidase and peptidase activities *in vitro* . FEBS Lett. 1995; 359: 73–77. 7851534 10.1016/0014-5793(94)01451-6

[21] Dayal S, Sparks A, Jacob J, Allende-Vega N, Lane DP, et al Suppression of the deubiquitinating enzyme USP5 causes the accumulation of unanchored polyubiquitin and the activation of p53. J Biol Chem. 2009; 284: 5030–5041. 10.1074/jbc.M805871200 19098288 PMC2696100

[22] Nakajima S, Lan L, Wei L, Hsieh CL, Rapić-Otrin V, Yasui A, et al Ubiquitin-specific protease 5 is required for the efficient repair of DNA double-strand breaks. PLoS One. 2014; 9:1 Available: http://www.plosone.org/article/info%3Adoi%2F10.1371%2Fjournal.pone.0084899. 10.1371/journal.pone.0084899 PMC3891734 24454762

[23] Tsou WL, Sheedlo MJ, Morrow ME, Blount JR, McGregor KM, Das C, et al Systematic analysis of the physiological importance of deubiquitinating enzymes. PLoS One. 2012; 7:8 Available: http://www.plosone.org/article/info%3Adoi%2F10.1371%2Fjournal.pone.0043112. 10.1371/journal.pone.0043112 PMC3427330 22937016

[24] Fan X, Huang Q, Ye X, Lin Y, Chen Y, Lin X, et al Drosophila USP5 controls the activation of apoptosis and the Jun N-terminal kinase pathway during eye development. PLoS One. 2014; 9:3 Available: http://www.plosone.org/article/info%3Adoi%2F10.1371%2Fjournal.pone.0092250. 10.1371/journal.pone.0092250 PMC3958489 24643212

[25] Morris EJ, Michaud WA, Ji JY, Moon NS, Rocco JW, Dyson NJ. Functional identification of Api5 as a suppressor of E2F-dependent apoptosis *in vivo* . PLoS Genet. 2006; 2:11 Available: http://www.plosgenetics.org/article/info%3Adoi%2F10.1371%2Fjournal.pgen.0020196. 10.1371/journal.pgen.0020196 PMC1636698 17112319

[26] Braid LR, Verheyen EM. Drosophila nemo promotes eye specification directed by the retinal determination gene network. Genetics. 2008; 180:283–299. 10.1534/genetics.108.092155 18757943 PMC2535682

[27] Fan Y, Wang S, Hernandez J, Yenigun VB, Hertlein G, Fogarty CE, et al Genetic models of apoptosis-induced proliferation decipher activation of JNK and identify a requirement of EGFR signaling for tissue regenerative responses in Drosophila. PLoS Genet. 2014; 10:1 Available: http://www.plosgenetics.org/article/info%3Adoi%2F10.1371%2Fjournal.pgen.1004131. 10.1371/journal.pgen.1004131 PMC3907308 24497843

[28] Verma R, Aravind L, Oania R, McDonald WH, Yates JR, Koonin EV, et al Role of Rpn11 metalloprotease in deubiquitination and degradation by the 26S proteasome. Science. 2002; 298: 611–615. 12183636 10.1126/science.1075898

[29] Reyes-Turcu FE, Horton JR, Mullally JE, Heroux A, Cheng X, Wilkinson KD. The ubiquitin binding domain ZnF UBP recognizes the C-terminal diglycine motif of unanchored ubiquitin. Cell. 2006; 124: 1197–1208. 16564012 10.1016/j.cell.2006.02.038

[30] Kaiser SE, Riley BE, Shaler TA, Trevino RS, Becker CH, Schulman H, et al Protein standard absolute quantification (PSAQ) method for the measurement of cellular ubiquitin pools. Nat Methods.2011; 8: 691–696. 10.1038/nmeth.1649 21743460 PMC3196335

[31] Oh C, Yoon JH, Park S, Yoo YJ. Simultaneous quantification of total and conjugated ubiquitin levels in a single immunoblot. Anal Biochem. 2013; 443: 153–155. 10.1016/j.ab.2013.09.011 24050967

[32] Wilkinson KD. Regulation of ubiquitin-dependent processes by deubiquitinating enzymes. FASEB J. 1997; 11: 1245–1256. 9409543 10.1096/fasebj.11.14.9409543

[33] Larsen CN, Krantz BA, Wilkinson KD. Substrate specificity of deubiquitinating enzymes: ubiquitin C-terminal hydrolases. Biochemistry. 1998; 37: 3358–3368. 9521656 10.1021/bi972274d

[34] Junn E, Ronchetti RD, Quezado MM, Kim SY, Mouradian MM. Tissue transglutaminase-induced aggregation of alpha-synuclein: Implications for Lewy body formation in Parkinson's disease and dementia with Lewy bodies. Proc Natl Acad Sci U S A.2003; 100: 2047–2052. 12576551 10.1073/pnas.0438021100 PMC149956

[35] Hanna J, Hathaway NA, Tone Y, Crosas B, Elsasser S, Kirkpatrick DS, et al Deubiquitinating enzyme Ubp6 functions noncatalytically to delay proteasomal degradation. Cel.2006; 127: 99–111. 10.1016/j.cell.2006.07.038 17018280

[36] Hanna J, Meides A, Zhang DP, Finley D. A ubiquitin stress response induces altered proteasome composition. Cell, 2007; 129: 747–759. 17512408 10.1016/j.cell.2007.03.042

[37] Hu M, Li P, Song L, Jeffrey PD, Chenova TA. Wilkinson KD et al Structure and mechanisms of the proteasome-associated deubiquitinating enzyme USP14. EMBO.2005; 24: 3747–3756. 16211010 10.1038/sj.emboj.7600832 PMC1276716

[38] Szlanka T, Haracska L, Kiss I, Deák P, Kurucz E, Andó I, et al Deletion of proteasomal subunit S5a/Rpn10/p54 causes lethality, multiple mitotic defects and overexpression of proteasomal genes in Drosophila melanogaster. J Cell Sci. 2003; 116: 1023–1033. 12584246 10.1242/jcs.00332

[39] Ju D, Wang L, Mao X, Xie Y. Homeostatic regulation of the proteasome via an Rpn4-dependent feedback circuit. Biochem Biophys Res Commun. 2004; 321: 51–57. 15358214 10.1016/j.bbrc.2004.06.105

[40] Chernova TA, Allen KD, Wesoloski LM, Shanks JR, Chernoff YO, Wilkinson KD. Pleiotropic effects of Ubp6 loss on drug sensitivities and yeast prion are due to depletion of the free ubiquitin pool. J Biol Chem. 2003; 278: 52102–52115. 14559899 10.1074/jbc.M310283200

[41] Hanna J, Leggett DS, Finley D. Ubiquitin depletion as a key mediator of toxicity by translational inhibitors. Mol Cell Biol. 2003; 23: 9251–9261. 14645527 10.1128/MCB.23.24.9251-9261.2003 PMC309641

[42] Xu D, Woodfield SE, Lee TV, Fan Y, Antonio C, Bergmann A. Genetic control of programmed cell death (apoptosis) in Drosophila. Fly(Austin). 2009; 3: 78–90. 19182545 10.4161/fly.3.1.7800 PMC2702463

[43] Holley CL, Olson MR, Colón-Ramos DA, Kornbluth S. Reaper eliminates IAP proteins through stimulated IAP degradation and generalized translational inhibition. Nat Cell Biol. 2002; 4:439–444. 12021770 10.1038/ncb798 PMC2713440

[44] Ryoo HD, Bergmann A, Gonen H, Ciechanover A, Steller H. Regulation of Drosophila IAP1 degradation and apoptosis by reaper and ubcD1. Nat Cell Biol. 2002; 4:432–438. 12021769 10.1038/ncb795

[45] Dantuma NP, Groothuis TA, Salomons FA, Neefjes J. A dynamic ubiquitin equilibrium couples proteasomal activity to chromatin remodeling. J Cell Biol. 2006; 173: 19–26. 16606690 10.1083/jcb.200510071 PMC2063781

[46] Kimura Y, Tanaka K. Regulatory mechanisms involved in the control of ubiquitin homeostasis. J Biochem. 2010; 147: 793–798. 10.1093/jb/mvq044 20418328

[47] Deveraux Q, Ustrell V, Pickart C, Rechsteiner M. A 26 S protease subunit that binds ubiquitin conjugates. J Biol Chem. 1994; 269: 7059–7061. 8125911

[48] Beal R, Deveraux Q, Xia G, Rechsteiner M, Pickart C. Surface hydrophobic residues of multiubiquitin chains essential for proteolytic targeting. Proc Natl Acad Sci. 1996; 93: 861–866. 8570649 10.1073/pnas.93.2.861 PMC40148

[49] Mimnaugh EG, Chen HY, Davie JR, Celis JE, Neckers L. Rapid deubiquitination of nucleosomal histones in human tumor cells caused by proteasome inhibitors and stress response inducers: effects on replication, transcription, translation, and the cellular stress response. Biochemistry. 1997; 36:14418–14429. 9398160 10.1021/bi970998j

[50] Wang H, Wang L, Erdjument-Bromage H, Vidal M, Tempst P, Jones RS, et al Role of histone H2A ubiquitination in Polycomb silencing. Nature. 2004; 431: 873–878. 15386022 10.1038/nature02985

[51] Wang CH, Chen GC, Chien CT. The deubiquitinase Leon/USP5 regulates ubiquitin homeostasis during Drosophila development. Biochem Biophys Res Commun. 2014; 452: 369–375. 10.1016/j.bbrc.2014.08.069 25152394

[52] Ecroyd H, Carver JA. Unraveling the mysteries of protein folding and misfolding. IUBMB Life. 2008; 60: 769–774. 10.1002/iub.117 18767168

[53] Bence NF, Sampat RM, Kopito RR. Impairment of the Ubiquitin-Proteasome System by Protein Aggregation. Science. 2001; 292: 1552–1555. 11375494 10.1126/science.292.5521.1552

[54] Snyder H, Mensah K, Theisler C, Lee J, Matouschek A, Wolozin B. Aggregated and monomeric alpha-synuclein bind to the S6’proteasomal protein and inhibit proteasomal function. J Biol Chem. 2003; 278: 11753–11759. 12551928 10.1074/jbc.M208641200

[55] Bainbridge SP, Bownes M. Staging the metamorphosis of Drosophila melanogaster. J Embryol Exp Morphol. 1981; 66: 57–80. 6802923

[56] Oh SW, Kingsley T, Shin HH, Zheng Z, Chen HW, Chen X, et al A P-element insertion screen identified mutations in 455 novel essential genes in Drosophila. Genetics. 2003; 163: 195–201. 12586707 10.1093/genetics/163.1.195 PMC1462436

[57] Pál M, Nagy O, Ménesi D, Udvardy A, Deák P. Structurally related TPR subunits contribute differently to the function of the anaphase-promoting complex in Drosophila melanogaster. J Cell Sci, 2007; 120: 3238–3248. 17878237 10.1242/jcs.004762

