# Supplementary figures and images for: Role of the Deubiquitylating Enzyme DmUsp5 in Coupling Ubiquitin Equilibrium to Development and Apoptosis in Drosophila melanogaster
Source: PLoS One. 2015 Mar 25;10(3):e0120875. doi: 10.1371/journal.pone.0120875 (PMC4373725; doi:10.1371/journal.pone.0120875)

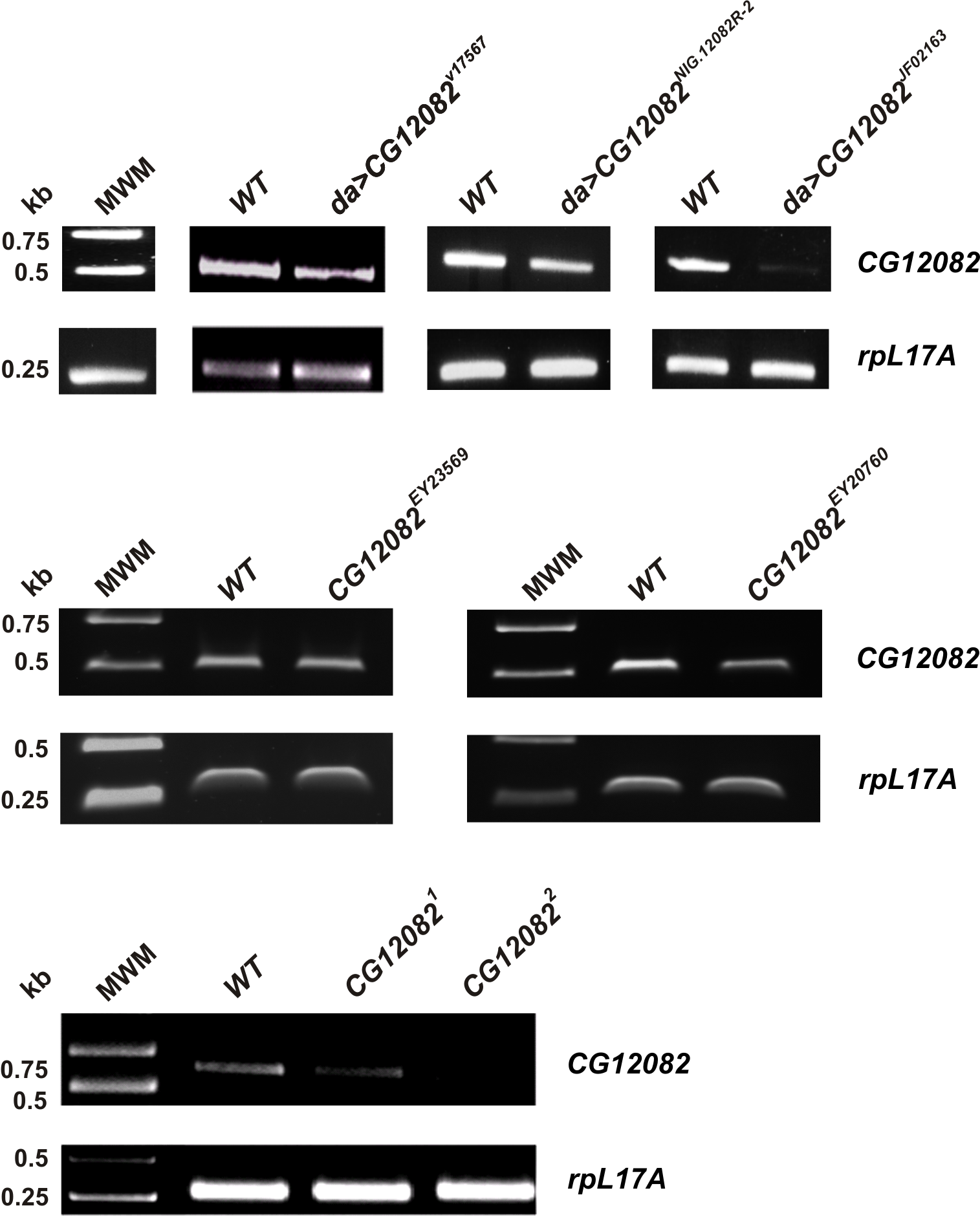

Supplement: S1 Fig — Expression levels were determined by semiquantitative RT-PCR followed by agarose gel electrophoresis. Samples were normalized to RpL17A expression. (TIF) [file pone.0120875.s001.tif]

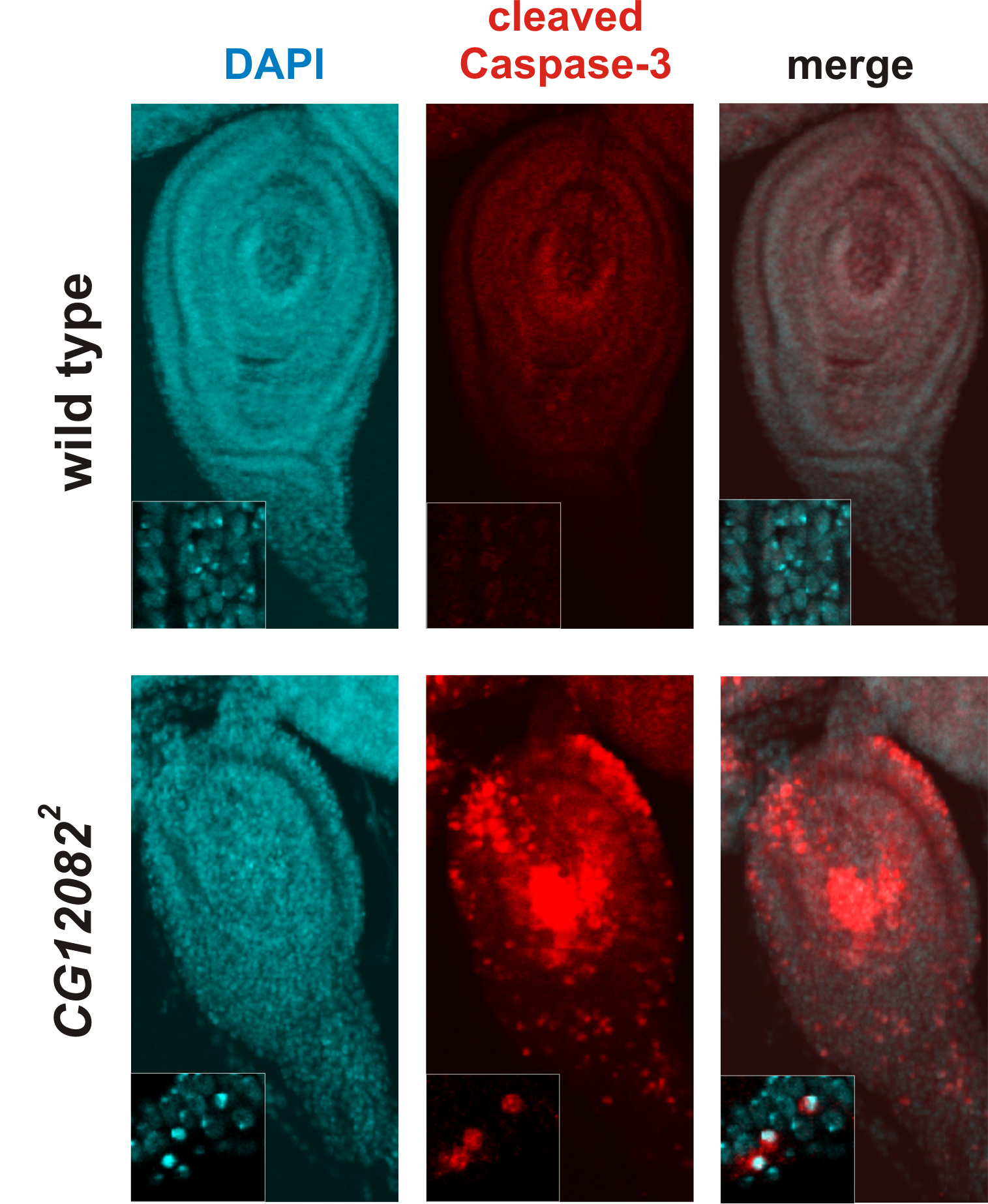

Supplement: S2 Fig — Leg discs of late L3 instar larvae from wild type (upper row) and CG12082 2 (lower row) were stained with DAPI (blue) and anti-cleaved caspase-3 antibody (red). Insets show individual cells at higher magnification. (TIF) [file pone.0120875.s002.tif]

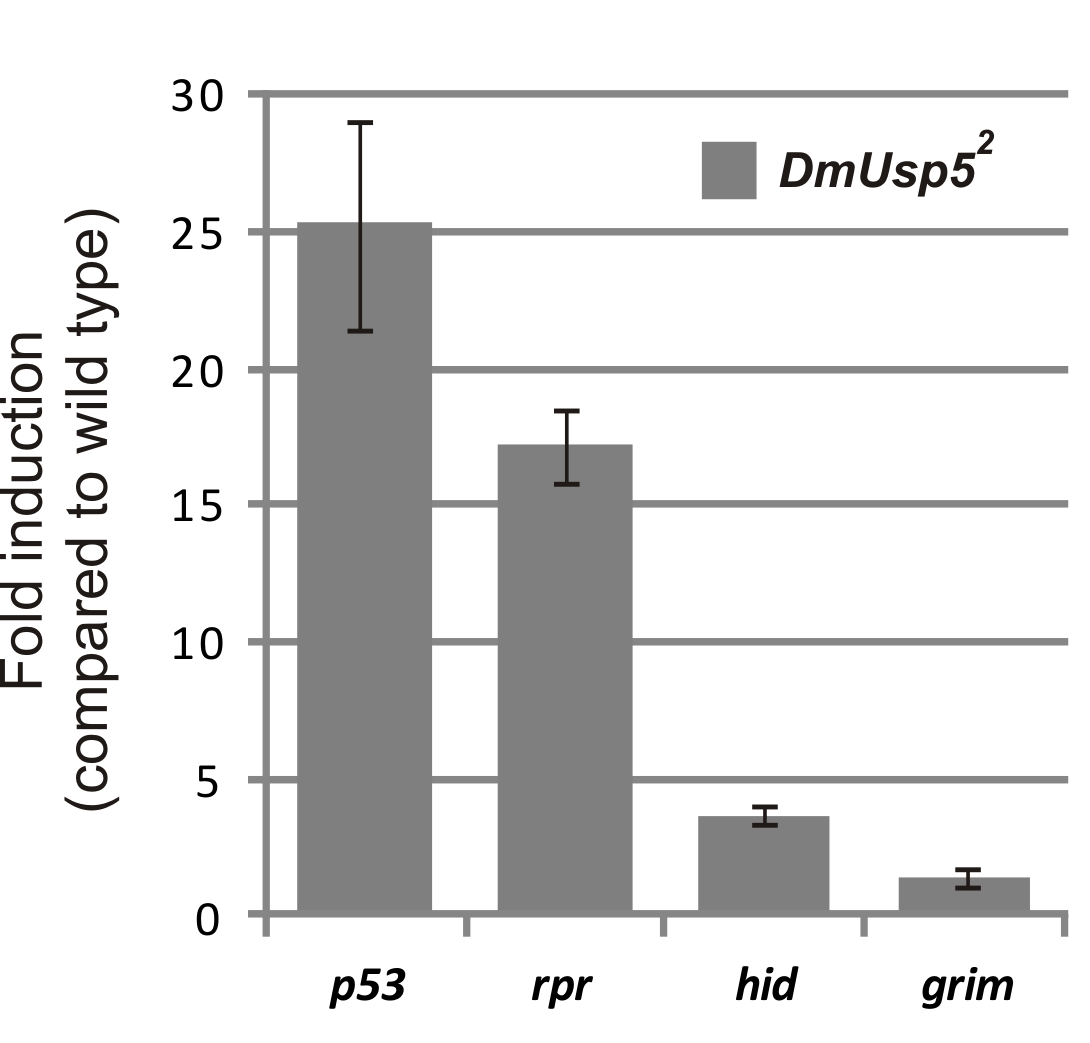

Supplement: S4 Fig — Total RNA was extracted from wild type and DmUsp5 2 larvae and reverse transcribed into cDNA. Quantitative real time PCR was performed on each pro-apoptotic gene normalized to Actin42A and rpL17A housekeeping genes. Columns represent the fold changes of gene expressions in DmUsp5 2 compared to wild type levels. Data represent mean and standard deviation of two independent experiments. (TIF) [file pone.0120875.s004.tif]

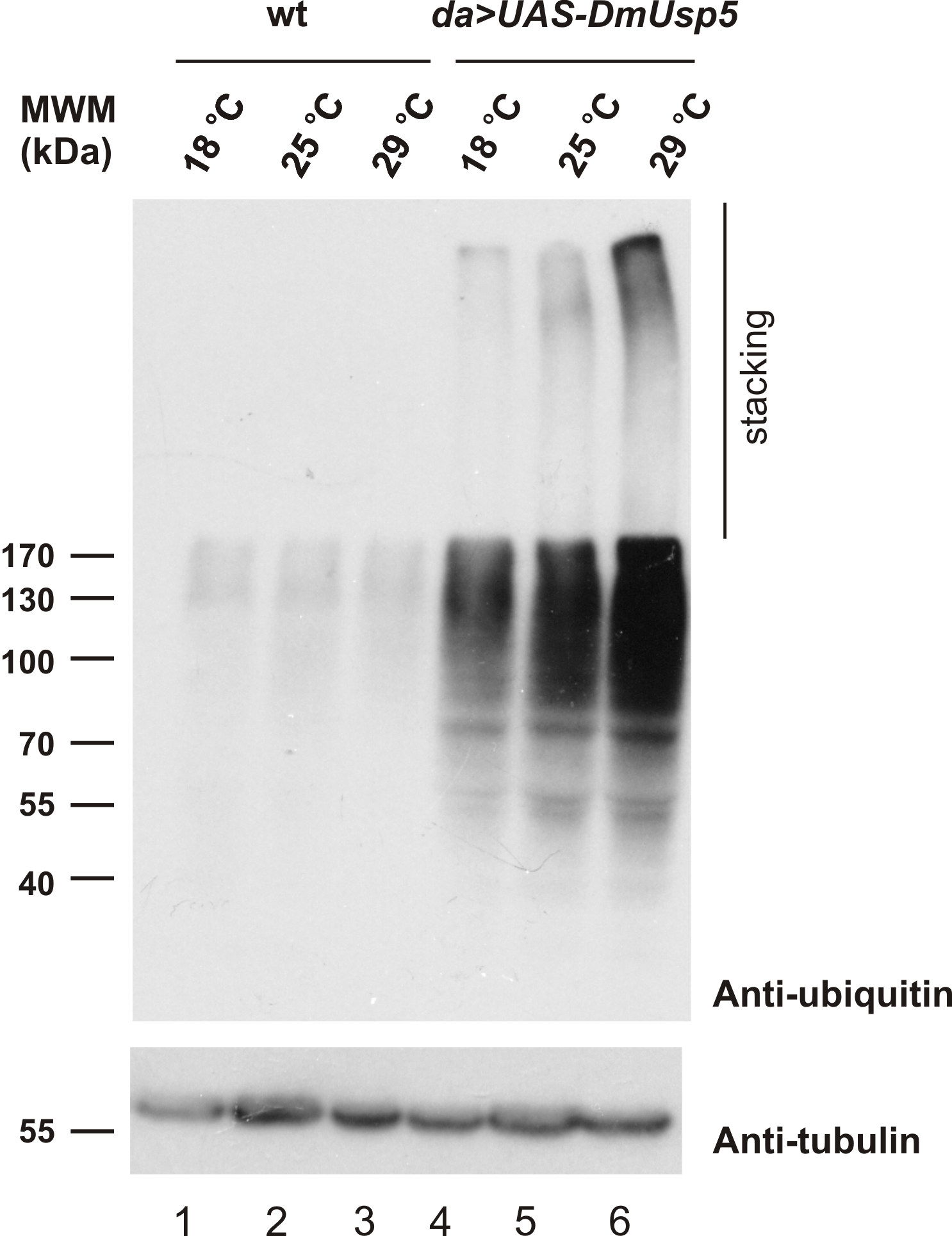

Supplement: S5 Fig — Protein extracts were prepared from third-instar larvae overexpressing DmUsp5 at the indicated temperatures. Samples were separated in an 8% SDS-PAGE gel, blotted onto a PVDF membrane and immunostained with a polyclonal anti-ubiquitin primary antibody. The Western blot reveals a fraction of high molecular weight ubiquitin species trapped in the stacking gel. (TIF) [file pone.0120875.s005.tif]
