# Supplementary material for: Role of the Deubiquitylating Enzyme DmUsp5 in Coupling Ubiquitin Equilibrium to Development and Apoptosis in Drosophila melanogaster
Source: PLoS One. 2015 Mar 25;10(3):e0120875. doi: 10.1371/journal.pone.0120875 (PMC4373725; doi:10.1371/journal.pone.0120875)
Supplement: S3 Fig — Amino acid sequences were aligned using Align X tool of Vector NTI 10 software. Conserved and similar amino acids are highlighted by red and yellow backgrounds, respectively. Black underline indicates the ubiquitin-like (UBL) domain. Catalytic cysteine and histidine residues are marked by black arrows. (PDF) [file pone.0120875.s003.pdf]

```
Dm Usp14   (1)  --MPAFKVKVKWGRELYTDIVVNTDEEPILFKAQLFALTGVQPDRQKVMCKGGILKDDQW
Hs USP14   (1)  --MPLYSVTVKWGKEKFEGVELNTDEPPMVFKAQLFALTGVQPARQKVMVKGGTLKDDDW
Sc Ubp6    (1)  MSGETFEFNIRHSGK-VYPITLSTDATSADLKSKAEELTQVPSARQKYMVKGGLSGEESI
                                          UBL

Dm Usp14  (59)  N--LQIKDGAVVLLLGSKES-VPEVPATPVKFIEDMNEAE-AATAMRLPAGLTNLGNTCY
Hs USP14  (59)  GN-IKIKNGMTLLMMGSADA-LPEEPSAKTVFVEDMTEEQ-LASAMELPCGLTNLGNTCY
Sc Ubp6   (60)  KIYPLIKPGSTVMLLGTPDANLISKPAKKNNFIEDLAPEQQVQQFAQLPVGFKNMGNTCY
                      UBL

Dm Usp14 (115)  MNATVQCLNAVPELRTALSTFSNDG--TDTMST----AFSISSAMKSIFAQM-EKG-TTV
Hs USP14 (116)  MNATVQCIRSVPELKDALKRYAGALRASGEMAS----AQYITAALRDLFDSM-DKTSSSI
Sc Ubp6  (120)  LNATLQALYRVNDLRDMILNYNPSQGVSNSGAQDEEIHKQIVIEMKRCFENLQNKSFKSV

Dm Usp14 (167)  TPIVLLQALHRASPQFAQTGENG-TYRQQDANECWAEILKMLQQKLRPKNQEP-------
Hs USP14 (171)  PPIILLQFLHMAFPQFAEKGEQG-QYLQQDANECWIQMMRVLQQKLEAIEDDSVKETDSS
Sc Ubp6  (169)  LPIVLLNTLRKCYPQFAERDSQGGFYKQQDAEELFTQLFHSMSIVFGDK-----------

Dm Usp14 (219)  --SNTVQKRHSSFIDQFFGGTFEVKMSSEEDPDEPSTVTSENFLQLSCFISMDVKYMQSG
Hs USP14 (230)  SASAATPSKKKSLIDQFFGVEFETTMKCTESEEEEVTKGKENQLQLSCFINQEVKYLFTG
Sc Ubp6  (218)  -------------FSEDFRIQFKTTIKDTANDNDITVKENESDSKLQCHISGTTNFMRNG

Dm Usp14 (277)  LKSKMKEQLVKKSETLGRDAKYIRTYLVSRLPAYLTVQFVRFQYKGKEGINAKVLKDIKF
Hs USP14 (290)  LKLRLQEEITKQSPTLQRNALYIKSSKISRLPAYLTIQMVRFFYKEKESVNAKVLKDVKF
Sc Ubp6  (265)  LLEGLNEKIEKRSDLTGANSIYSVEKKISRLPKFLTVQYVRFFWKRSTNKKSKILRKVVF

Dm Usp14 (337)  PIDFDAFELCTPELQNKLCPMRSKFKDLEDKKMEVDVVKRNEPN---------EE-----
Hs USP14 (350)  PLMLDMYELCTPELQEKMVSFRSKFKDLEDKKVNQQPNTSDKKS---------SP-----
Sc Ubp6  (325)  PFQLDVADMLTPEYAAEKVKVRDELRKVEKEKNEKEREIKRRKFDPSSSENVMTPREQYE

Dm Usp14 (383)  --------KKDV----KYEQFWFDDDLGSNNSGYYTLQAVLTHKGRSSSSGHYVAWVRSS
Hs USP14 (396)  --------QKEV----KYEPFSFADDIGSNNCGYYDLQAVLTHQGRSSSSGHYVSWVKRK
Sc Ubp6  (385)  TQVALNESEKDQWLEEYKKHFPPNLEKGENPSCVYNLIGVITHQGANSESGHYQAFIRDE

Dm Usp14 (431)  --GDVWFKFDDDEVSAVATDEILRLSGGGDWHCAYVLLYAPRRLEKL------
Hs USP14 (444)  --QDEWIKFDDDKVSIVTPEDILRLSGGGDWHIAYVLLYGPRRVEIMEEESEQ
Sc Ubp6   (445) LDENKWYKFNDDKVSVVEKEKIESLAGGGESDSALILMYKGFGL---------
```
